# Common questions and misconceptions about creatine supplementation: what does the scientific evidence really show?

**DOI:** 10.1186/s12970-021-00412-w

**Published:** 2021-02-08

**Authors:** Jose Antonio, Darren G. Candow, Scott C. Forbes, Bruno Gualano, Andrew R. Jagim, Richard B. Kreider, Eric S. Rawson, Abbie E. Smith-Ryan, Trisha A. VanDusseldorp, Darryn S. Willoughby, Tim N. Ziegenfuss

**Affiliations:** 1 Department of Health and Human Performance, Nova Southeastern University, Davie, Florida USA; 2 Faculty of Kinesiology and Health Studies, University of Regina, Regina, Canada; 3 Department of Physical Education, Faculty of Education, Brandon University, Brandon, MB Canada; 4 Applied Physiology & Nutrition Research Group; School of Medicine, FMUSP, University of Sao Paulo, Sao Paulo, SP Brazil; 5 Sports Medicine Department, Mayo Clinic Health System, La Crosse, WI USA; 6 Exercise & Sport Nutrition Lab, Human Clinical Research Facility, Department of Health & Kinesiology, Texas A&M University, College Station, USA; 7 Department of Health, Nutrition, and Exercise Science, Messiah University, Mechanicsburg, PA USA; 8 Department of Exercise and Sport Science, University of North Carolina, Chapel Hill, NC USA; 9 Department of Exercise Science and Sport Management, Kennesaw State University, Kennesaw, GA USA; 10 School of Exercise and Sport Science, University of Mary Hardin-Baylor, Belton, TX USA; 11 The Center for Applied Health Sciences, Canfield, Ohio USA

**Keywords:** Social Media, Anecdotal, Research, Adverse Effects, Safety

## Abstract

Supplementing with creatine is very popular amongst athletes and exercising individuals for improving muscle mass, performance and recovery. Accumulating evidence also suggests that creatine supplementation produces a variety of beneficial effects in older and patient populations. Furthermore, evidence-based research shows that creatine supplementation is relatively well tolerated, especially at recommended dosages (i.e. 3-5 g/day or 0.1 g/kg of body mass/day). Although there are over 500 peer-refereed publications involving creatine supplementation, it is somewhat surprising that questions regarding the efficacy and safety of creatine still remain. These include, but are not limited to: 1. Does creatine lead to water retention? 2. Is creatine an anabolic steroid? 3. Does creatine cause kidney damage/renal dysfunction? 4. Does creatine cause hair loss / baldness? 5. Does creatine lead to dehydration and muscle cramping? 6. Is creatine harmful for children and adolescents? 7. Does creatine increase fat mass? 8. Is a creatine ‘loading-phase’ required? 9. Is creatine beneficial for older adults? 10. Is creatine only useful for resistance / power type activities? 11. Is creatine only effective for males? 12. Are other forms of creatine similar or superior to monohydrate and is creatine stable in solutions/beverages? To answer these questions, an internationally renowned team of research experts was formed to perform an evidence-based scientific evaluation of the literature regarding creatine supplementation.

## Introduction

Creatine (methylguanidine-acetic acid) is endogenously formed from reactions involving the amino acids arginine, glycine and methionine in the kidneys and liver [1]. Exogenously, creatine is primarily consumed from meat and/or as a dietary supplement. According to PubMed (archive of biomedical and life sciences journal literature at the U.S. National Institutes of Health’s National Library of Medicine) there are over 500 peer-refereed publications involving various aspects of creatine supplementation. Based on the enormous popularity of creatine supplementation, the International Society of Sports Nutrition (ISSN) published an updated position stand in 2017 on the safety and efficacy of creatine supplementation in exercise, sport, and medicine [2]. This comprehensive paper provided an evidence-based review of the literature examining the effects of creatine supplementation on performance, recovery, injury prevention, exercise tolerance and rehabilitation, neuroprotection, aging, clinical and disease state populations, and pregnancy. Importantly, the safety profile of creatine was also reviewed. As of September 1, 2020, the paper has been viewed 179,000 times and cited 100 times (according to Web of Science). Furthermore, Altmetric data indicates that the paper has been mentioned in 19 news outlets, 4 blogs, 492 tweets, 54 Facebook pages, and been uploaded 69 times in video posts. Instagram stories and posts are not included as Altmetric data.

Despite the widespread outreach of the 2017 ISSN position stand paper [2], along with other evidence-based review/meta-analysis papers involving various aspects of creatine supplementation published after the 2015 Creatine in Health, Sport and Medicine Conference in Germany [3–34], questions and misconceptions involving creatine supplementation still remain. These include, but are not limited to: 1. Does creatine supplementation lead to water retention? 2. Is creatine is an anabolic steroid? 3. Does creatine supplementation cause kidney damage / renal dysfunction? 4. Does creatine supplementation cause hair loss / baldness? 5. Does creatine supplementation lead to dehydration and muscle cramping? 6. Is creatine supplementation harmful for children and adolescents? 7. Does creatine supplementation increase body fat? 8. Is a creatine supplementation ‘loading-phase’ required? 9. Is creatine supplementation beneficial for older adults? 10. Is creatine supplementation only useful for resistance/power type activities? 11. Is creatine supplementation only effective for males? 12. Are other forms of creatine similar or superior to monohydrate? Is creatine stable in solutions/beverages? To address these questions, an internationally renowned team of research experts, who have collectively published over 200 peer-refereed articles involving creatine supplementation, was formed to perform an evidence-based scientific evaluation of the literature. Each question was answered by one researcher, chosen according to her/his expertise on the topic. Then, the final version of this manuscript was reviewed and approved by all authors, therefore reflecting the group opinion.

### Does creatine lead to water retention?

The purported myth of creatine supplementation increasing body water (TBW) is likely due to early research which showed that creatine supplementation at 20 g/day for six days was associated with water retention [35]. It does appear that the most common adverse effect of creatine supplementation is water retention in the early stages (first several days) [36]. For example, studies have shown that three days of creatine supplementation increased TBW and extracellular body water (ECW) [37] and intracellular water (ICW) [38]. Unfortunately, based on these short-term responses, this notion that creatine increases water retention over the long-term has been widely accepted [39].

Creatine is an osmotically active substance. Thus, an increase in the body's creatine content could theoretically result in increased water retention. Creatine is taken up into muscle from circulation by a sodium-dependent creatine transporter [1]. Since the transport involves sodium, water will also be taken up into muscle to help maintain intracellular osmolality. However, considering the activity of the sodium-potassium pumps, it is not likely that intracellular sodium concentration is dramatically affected by creatine supplementation [39].

A number of exercise training studies (e.g., 5-10 weeks) incorporating creatine supplementation have shown no increases in total body water (TBW). For example, resistance-trained males who received creatine at a dose of 0.3 g/kg lean body mass/day for 7 days (approximately 20 g/day) followed by 4 weeks at 0.075 g/kg lean body mass/day for 28 days (approximately 5 g/day) experienced no significant change in ICW, ECW, or TBW [40]. Furthermore, resistance-trained males who consumed creatine supplementation (20 g/day for seven days followed by 5 g/day for 21 days) had no significant increase in ICW, ECW, or TBW [41]. Similarly, males and females ingesting creatine (0.03 g/kg/day for six weeks) experienced no significant increase in TBW [42]. Six weeks of creatine supplementation in non-resistance-trained males at a dosage of 0.3 g/kg lean body mass for five days followed by 0.075 g/kg lean body mass for 42 days produced no significant changes in TBW [43]. In contrast, when assessing TBW, ICW, and ECW content before and after 28 days of creatine supplementation in healthy males and females (n = 32), Powers et al. [44] showed that creatine supplementation was effective at increasing muscle creatine content which was associated with an increase in body mass and TBW but did not alter ICW or ECW volumes. In a recent study examining the effects of creatine supplementation combined with resistance exercise for 8 weeks, Ribeiro et al. [45] found a significant increase in TBW (7.0%) and ICW (9.2%) volume compared to placebo (TBW: 1.7%; ICW: 1.6%), with both groups similarly increasing ECW (CR: 1.2% vs. Placebo = 0.6%). Importantly, the ratio of skeletal muscle mass to ICW remained similar in both groups. It is important to highlight that the ICW is an important cellular signal for protein synthesis and thus drives an increase in muscle mass over time [46].

***In summary, while there is some evidence to suggest that creatine supplementation increases water retention, primarily attributed to increases in intracellular volume, over the short term, there are several other studies suggesting it does not alter total body water (intra or extracellular) relative to muscle mass over longer periods of time. As a result, creatine supplementation may not lead to water retention.***

### Is creatine an anabolic steroid?

Anabolic steroids are a synthetic version of testosterone, an androgenic hormone which is also produced endogenously within both males and females, and is used in conjunction with resistance training with the intent of enhancing muscle mass and strength due to increases in muscle protein synthesis [47]. This increase in MPS is due to testosterone’s ability to enter the muscle cell, bind with the intracellular androgen receptor, and increase the expression of various muscle-specific genes [48]. Creatine is converted to phosphocreatine (PCr), regulated by the enzyme creatine kinase (CK) in muscle and used to create intracellular adenosine triphosphate (ATP) production [1]. Creatine supplementation, however, can increase the capacity of ATP and energy produced during heavy anaerobically-related exercise, thereby possibly increasing muscle power, repetitions and exercise volume which can subsequently contribute to muscle performance and hypertrophy over the course of a training period [2].

While the physiological and performance outcomes of anabolic steroids and creatine can be similar, their mechanisms of action and legal categorization are not. Anabolic steroids are drugs, with a different chemical structure than creatine, and are Class C, Schedule III controlled substances regulated by the Food and Drug Administration (FDA) and subject to the regulatory control provisions of the Controlled Substances Act (CSA) set forth by the Drug Enforcement Association (DEA). Creatine, on the other hand, like many other dietary supplements fits well within the confines of The Dietary Supplement Health and Education Act of 1994 ("DSHEA"), which is a statute of United States Federal legislation which defines and regulates dietary supplements by the Federal Drug Administration (FDA) for Good Manufacturing Practices (GMP). It is illegal to possess and administer anabolic steroids without a physician’s prescription. However, there are no legal ramifications for the possession or ingestion of creatine.

***In summary, because creatine has a completely different chemical structure, it is not an anabolic steroid.***

### Does creatine cause kidney damage/renal dysfunction?

Questions and concerns involving creatine supplementation and kidney damage/renal dysfunction are common. In terms of pervasive misinformation in the sport nutrition arena, the notion that creatine supplementation leads to kidney damage/renal dysfunction is perhaps second only to the myth that protein supplementation and high habitual protein intake causes kidney damage. Today, after > 20 years of research which demonstrates no adverse effects from recommended dosages of creatine supplements on kidney health, unfortunately, this concern persists. While the origin is unknown, the connection between creatine supplementation and kidney damage/renal dysfunction could be traced back to two things: a poor understanding of creatine and creatinine metabolism and a case study published in 1998.

In skeletal muscle, both creatine and PCr are degraded non-enzymatically to creatinine, which is exported to the blood and excreted in the urine [1]. Healthy kidneys filter creatinine, which would otherwise increase in the blood. Therefore, blood creatinine levels can be used as a proxy marker of kidney function. However, the amount of creatinine in the blood is related to muscle mass (i.e. males have higher blood creatinine than females) and both dietary creatine and creatinine intake [35]. Both blood and urinary creatinine may be increased by ingestion of creatine supplementation and creatine containing foods, such as meat. Creatine is normally not present in urine, but can reach very high levels (>10 g/day) during creatine supplementation [49]. There appears to be an unsubstantiated perspective that if the kidneys are “forced” to excrete higher than normal levels of creatine or creatinine, some sort of kidney “overload” will take place, causing kidney damage and/or renal dysfunction. In reality, transient increases in blood or urinary creatine or creatinine due to creatine supplementation are unlikely to reflect a decrease in kidney function. Additionally, one must exercise caution when using blood creatinine and estimated creatinine clearance/glomerular filtration rate in individuals who consume high meat intake or supplement with creatine. In a review of creatine supplementation studies, Persky and Rawson [50] found no increase in serum creatinine in 12 studies, 8 studies showed an increase that remained within the normal range, and only 2 studies showed an increase above normal limits (although not different from the control group in one study).

In 1998, a case study of a young male with focal segmental glomerulosclerosis and relapsing nephrotic syndrome was reported [51]. The young male, who had kidney disease for 8 years and was treated with cyclosporine (i.e., immunosuppressant) for 5 years, had recently begun ingesting creatine supplementation (15 g/day for 7 days; followed by 2 g/day for 7 weeks). Based on increased blood levels of creatinine and subsequent estimate of calculated creatinine clearance, his kidney health was presumed to be deteriorating, although he was otherwise in good health. The patient was encouraged to discontinue creatine supplementation. At this time, it was already known that blood and urine creatinine levels can increase following ingestion of creatine containing food products, including creatine supplements [35]. This was ignored by the authors of this case study, as was the inclusion of two investigations which demonstrated that creatine supplementation did not negatively impact renal function [52, 53]. The dosage of creatine during the maintenance phase, which was also ignored, was only slightly higher than the daily creatine intake of a typical omnivore’s dietary intake, or in terms of food, a large hamburger or steak per day (meat contains about 0.7 g of creatine / 6 oz. serving; see [54]). In response to this case study, two separate teams of experts in creatine metabolism wrote letters to the editor of Lancet [53, 55]. However, the notion that creatine supplementation leads to kidney damage and/or renal dysfunction gained traction and momentum.

Since this case study was reported in 1998, experimental and controlled research trials investigating the effects of creatine supplementation on kidney/renal function has substantially increased [50, 56–58]. Overall, in healthy individuals, there appears to be no adverse effects from consuming recommended doses of creatine supplements on kidney/renal function [50, 56–58]. Interestingly, Gualano et al. [58] reviewed a small number of case studies which reported renal dysfunction in individuals who were supplementing with creatine. Similar to the case report by Pritchard and Kalra [51], these additional case reports were confounded by medications, pre-existing kidney disease, concomitant supplement ingestion, inappropriate creatine dosages (e.g., 100 X recommended dose), and anabolic androgenic steroid use.

It is prudent to be cautious when ingesting any dietary supplement or medication. Survey data indicates that creatine supplementation usage ranges between 8-74% in athletes and other exercising individuals (reviewed in Rawson et al. [59]). Even with a low estimate of 8% of exercising individuals using creatine supplements, this indicates thousands of exposures across several decades. If the link between creatine supplementation and kidney health was valid, there would be an expected increase in kidney damage / renal dysfunction in low risk (i.e. young, physically fit, healthy) individuals since 1992 after Harris et al. published their seminal work [60]. After nearly 30 years of post-marketing surveillance, thousands of exposures, and multiple clinical trials, no such evidence exists.

***In summary, experimental and controlled research indicates that creatine supplementation, when ingested at recommended dosages, does not result in kidney damage and/or renal dysfunction in healthy individuals.***

### Does creatine cause hair loss / baldness?

The vast majority of speculation regarding the relationship between creatine supplementation and hair loss/baldness stems from a single study by van der Merwe et al. [61] where college-aged male rugby players who supplemented with creatine (25 g/day for 7 days, followed by 5 g/day thereafter for an additional 14 days) experienced an increase in serum dihydrotestosterone (DHT) concentrations over time. Specifically, DHT increased by 56% after the seven-day loading period, and remained 40% above baseline values after the 14-day maintenance period. These results were statistically significant compared to when the subjects consumed a placebo (50 g of glucose per day for 7 days, followed by 30 g/day for 14 days thereafter). Given that changes in these hormones, particularly DHT, have been linked to some (but not all) occurrences of hair loss/baldness [62], the theory that creatine supplementation leads to hair loss / baldness gained some momentum and this potential link continues to be a common question / myth today. It is important to note that the results of van der Merwe et al. [61] have not been replicated, and that intense resistance exercise itself can cause increases in these androgenic hormones.

DHT is a metabolite of testosterone, formed when the enzyme 5-alpha-reductase converts free testosterone to DHT [63]. In males, DHT can bind to androgen receptors in susceptible hair follicles and cause them to shrink, ultimately leading to hair loss [64]. However, in the van der Merwe et al. [61] study, no increase in total testosterone was found in the 16 males who completed the study. Free testosterone was not measured. Moreover, the increase in DHT and the DHT: testosterone ratio remained well within normal clinical limits. Furthermore, baseline (prior to supplementation), DHT was 23% lower in the creatine group (0.98 nmol/L) compared to the placebo group (1.26 nmol/L). Thus the small increase in DHT in the creatine group (+ 0.55 nmol/L after 7 days of supplementation and + 0.40 nmol/L after 21 days of supplementation), in combination with a small decrease in the placebo DHT response (-0.17 nmol/L after 7 days of supplementation and -0.20 nmol/L after 21 days of supplementation) explains the “statistically significant” increase in DHT noted by van der Merwe et al. [61]. While it is possible that creatine supplementation upregulated 5-alpha-reductase activity in these males (potentially leading to increased formation of DHT), no study has reported hair loss/baldness in humans.

To date, 12 other studies have investigated the effects of creatine supplementation (i.e. doses ranging from 3-25 g/day for 6 days to 12 weeks) on testosterone. Two studies reported small, physiologically insignificant increases in total testosterone after six and seven days of supplementation [65, 66], while the remaining ten studies reported no change in testosterone concentrations. In five of these studies [67–71], free testosterone, which the body uses to produce DHT, was also measured and no increases were found.

***In summary, the current body of evidence does not indicate that creatine supplementation increases total testosterone, free testosterone, DHT or causes hair loss/baldness.***

### Does creatine lead to dehydration and muscle cramping?

Speculation exists that creatine supplementation causes dehydration and muscle cramping [72, 73]. In the early 2000’s, with limited data and based primarily on speculation, the American College of Sports Medicine (ACSM) recommended that individuals controlling their weight and exercising intensely or in hot environments should avoid the use of creatine supplementation [74]. The physiological rationale suggesting that creatine supplementation may cause dehydration and muscle cramping is based on the premise that creatine is an osmotically active substance found primarily in skeletal muscle and may alter whole-body fluid distribution by preferentially increasing intracellular water uptake and retention, particularly over the short-term [38, 75]. In situations of body water loss, such as severe sweating from exercise and/or increased environmental temperature, the bound intracellular fluid, in theory, may be detrimental to thermal regulation and lead to extracellular dehydration, electrolyte imbalance and muscle cramping or other heat-related musculoskeletal issues [44]. The initial loading phase of creatine supplementation (i.e. 20 g/day for 5-7 days) typically results in a 1-3 kg increase in body mass, mostly attributable to net body water retention [75, 76]. Some anecdotal evidence indicates that creatine users perceive supplementation to result in some adverse effects [77]. For example, in a survey involving 219 athletes, 90 participants reported using creatine with 34 of them (38%) reporting perceived negative effects such as cramping (27%) [77]. Similarly, in National Collegiate Athletic Association (NCAA) Division 1 baseball and football players (N=52) using creatine, 25% reported incidences of muscle cramping and 13.5% reported symptoms of dehydration. Importantly, these studies failed to control for the use of other supplements and the dosage of creatine ingested. Greenwood et al. [77] noted that 91% of participants exceeded the recommended creatine maintenance dose of 5 g/day. However, these self-report surveys are in contradiction to experimental and clinical evidence. Greenwood et al. [78] monitored injury rates in Division IA NCAA collegiate football players (N=72; age: 19.7 ± 1.0 yrs) where environmental conditions were hot (27.3 ± 10.9^0^C) and humid (54.2 ± 9.7%). Participants chose to receive either creatine (n = 38: 0.3 g/kg/day for 5 days; followed by 0.03 g/kg/day for 115 days) or a sport drink placebo (n = 34) throughout the football season. Injuries treated by the athletic training staff were monitored. Creatine users had significantly less cramping (p = 0.021), heat illnesses and dehydration (p = 0.043), muscle tightness (p = 0.020), muscle strains (p = 0.021), and total injuries (p < 0.001) compared to non-users. Non-contact joint injuries, contact injuries, illnesses, missed practices due to injuries, and players lost for the season were not different between groups. In a clinical setting, haemodialysis patients (n = 10) who frequently reported muscle cramping were provided creatine (12 g) 5 minutes prior to haemodialysis [79]. Creatine supplementation reduced the frequency of symptomatic muscle cramping by 60% [79]. These beneficial effects from creatine may be explained by fluid distribution and electrolyte imbalances, as previously discussed.

***In summary, experimental and clinical research does not validate the notion that creatine supplementation causes dehydration and muscle cramping.***

### Is creatine harmful for children and adolescents?

Concerns regarding the safety of creatine supplementation in children and adolescents (< 19 yrs) continues to be highly prevalent. The overwhelming majority of evidence in adult populations indicates that creatine supplementation, both short- and longer-term, is safe and generally well tolerated [2]. However, the question of whether or not this holds true for children and adolescents is relatively unclear. The physiological rationale supporting the potential ergogenic benefits of creatine supplementation in children and adolescents was first postulated by Unnithan and colleagues in 2001 [80]; which established a strong basis for future applications of creatine for younger athletes. More recently, in a comprehensive review examining the safety of creatine supplementation in adolescents, Jagim et al. [16] summarized several studies that examined the efficacy of creatine supplementation among various adolescent athlete populations and found no evidence of adverse effects. However, it is important to note that none of the performance-focused studies included in the Jagim et al. [16] review provided data examining specific markers of clinical health and whether or not they were impacted by the supplementation protocols.

From a clinical perspective, creatine supplementation has been found to potentially offer health benefits with minimal adverse effects in younger populations. Hayashi et al. [81] found improvements in pediatric patients with systemic lupus erythematosus and reported no adverse changes in laboratory parameters of hematology, kidney function, liver function or inflammatory markers after 12 weeks of creatine supplementation. Tarnopolsky et al. [82] reported significant improvements in fat-free mass and hand grip strength in 30 pediatric patients with Duchenne muscular dystrophy following 4 months of creatine supplementation. Importantly, the creatine supplementation protocol appeared to be well tolerated and did not adversely affect laboratory markers of kidney function, oxidative stress, and bone health [81–83]. In addition, Sakellaris et al. [83] reported significant improvements in traumatic brain injury-related outcomes in children and adolescents who received oral creatine supplementation (0.4 g/kg/day) for 6 months. These neurological benefits may have potential applications for young athletes participating in collision sports, which pose underlying risks of concussions or sub-concussive impacts. Further, several of these clinical trials implemented strict clinical surveillance measures, including continual monitoring of laboratory markers of kidney health, inflammation, and liver function; none of which were negatively impacted by the respective creatine supplementation interventions. These findings support the hypothesis of creatine supplementation likely being safe for children and adolescents. However, perhaps the strongest supporting evidence for the safety of creatine is the recent classification of creatine as generally recognized as safe (GRAS) by the United States Food and Drug Administration (FDA) in late 2020 (https://www.fda.gov/media/143525/download). Ultimately, this classification indicates that the currently available scientific data pertaining to the safety of creatine, is sufficient and has been agreed upon by a consensus of qualified experts, thereby determining creatine to be safe under the conditions of its intended use (https://www.fda.gov/media/143525/download). Even though infants and young children are excluded from GRAS, this would still apply to older children and adolescent populations.

The majority of dietary supplement survey data indicates that a relatively high percentage of youth and adolescent athletes are currently or have previously supplemented with creatine. For example, Kayton et al. [84] found that in a sample of 270 high school boys and girls, 21% of boys and 3% of girls reported supplementing with creatine. Furthermore, in a sample of elite Olympic level sample of young German athletes (14-18 yrs), 12% of those surveyed reported supplementing with creatine [85]. Therefore, these trends warrant additional research to determine with greater certainly whether creatine supplementation, both acute and longer-term, is safe for children and adolescents.

***In summary, based on the limited evidence, creatine supplementation appears safe and potentially beneficial for children and adolescents.***

### Does creatine increase fat mass?

The theory that creatine supplementation increases fat mass is a concern amongst exercising individuals, possibly because some experience a gain in body mass from creatine supplementation. However, randomized controlled trials (one week to two years in duration) do not validate this claim. Acute creatine supplementation (7 days) had no effect on fat mass in young and older adults; however, fat-free mass was increased [86, 87]. Furthermore, three weeks of creatine supplementation had no effect on body composition in swimmers [88]. The addition of creatine to high-intensity interval training had no effect on body composition in recreationally active females [89]. In addition, the effects of creatine supplementation during resistance training overreaching had no effect on fat mass [70]. Moreover, in a group of healthy recreational male bodybuilders, 5 g/day of creatine consumed either pre- or post-training had no effect on fat mass [90]. In other short-terms studies lasting 6-8 weeks, there were no changes in fat mass from creatine supplementation. Becque et al. [91] found no changes in fat mass after six weeks of supplementation plus resistance training. In another 6-week investigation, no significant differences in fat mass or percentage body fat were observed after creatine supplementation [42]. Furthermore, creatine supplementation during an 8-week rugby union football season also had no effect on fat mass [92].

One might suggest that eight weeks or less of creatine supplementation is insufficient to arrive at a definitive conclusion regarding creatine’s effect on fat mass. Nonetheless, there are several investigations that have used much longer treatment periods. For example, healthy resistance-trained males were randomly assigned in a double-blind fashion to supplement with creatine (i.e., 20 g/day for 1 week followed by 5 g/day for 11 weeks) or placebo [93]. Lean body mass and muscle fiber size increased; percent body fat and fat mass were unaffected over the 12-week training period [93]. In older males (~70 yrs), 12 weeks of creatine supplementation during resistance training had no effect (compared to placebo) on fat mass [94]. Furthermore, Gualano et al. assessed the effects of creatine supplementation (24 weeks), with and without resistance training, in older females. Results showed no effect from creatine on fat mass [95]. Candow et al. [96] examined the effects of creatine supplementation in older adults (50-71 years) over a 32-week treatment period. Study participants were randomized to supplement with creatine or placebo before or after resistance training (3 days per week). There was an increase over time for lean tissue and strength with a decrease in fat mass. From a clinical perspective, children with acute lymphoblastic leukemia who supplemented with creatine (0.1 g/kg/day) for two sequential periods of 16 weeks experienced a significant reduction in fat mass. In contrast, the children who did not consume creatine gained fat mass [97]. In two studies involving postmenopausal women, Lobo et al. [98] found no change in absolute or relative body fat from one-year of low-dose creatine supplementation. Furthermore, two years of creatine supplementation also had no effect on fat mass [99].

Recently, Forbes et al. [100] conducted a systematic review and meta-analysis on randomized controlled trials involving creatine supplementation in conjunction with resistance training on fat mass in older adults (≥ 50 yrs). Nineteen studies with a total of 609 participants were included. Participants supplementing with creatine had a greater reduction in body fat percentage. There was no significant difference in absolute fat mass loss; however, the creatine group lost ~0.5 kg more fat mass compared to those on placebo.

***In summary, creatine supplementation does not increase fat mass across a variety of populations.***

### Is a creatine ‘loading-phase’ required?

Pioneering research in the early 1900’s using animal models showed that creatine supplementation could augment creatine content by 70% [101, 102]. Decades later, Harris et al. [60] published a seminal paper which showed that ‘loading’ with creatine increased skeletal muscle creatine stores, as evaluated from muscle biopsies collected from the vastus lateralis in young, healthy human participants. This research sparked incredible interest in studying creatine supplementation strategies that would increase intramuscular creatine content, helping shape current recommendations.

**Fig. 1:**
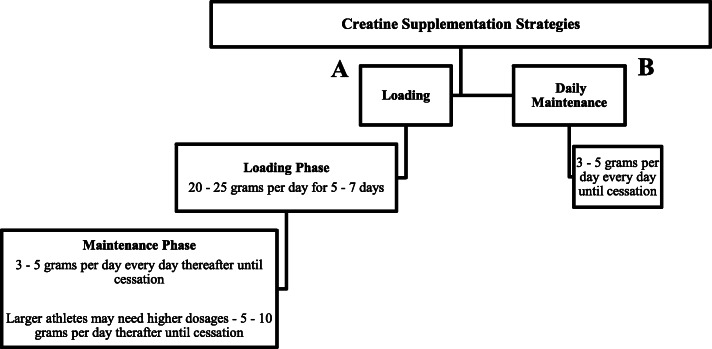
Creatine supplementation strategies.

Creatine ‘loading’ is defined as supplementing with oral creatine for 5–7 days with a dosage of 20–25 g/day, often divided into smaller doses throughout the day (e.g., four to five, 5 g servings/day). Creatine ‘loading’ may also be prescribed relative to body mass, for example, 0.3 g/kg/d for 5-7 days (i.e., 21 g/day for a 70 kg individual). The ‘loading’ phase of creatine supplementation is followed by a daily ‘maintenance’ phase often ranging from daily 3–5 g servings/day (Figure 1, side A). In addition to the seminal work of Harris et al. [60], several other investigations have demonstrated increased intramuscular creatine stores in humans from the creatine ‘loading’ phase [35, 103, 104]. A common misconception regarding creatine supplementation is that individuals must ‘load’ with creatine to increase intramuscular creatine stores and subsequently experience the purported ergogenic benefits of creatine supplementation. However, lower daily creatine supplementation dosing strategies (i.e., 3-5 g/day) are well established throughout the scientific literature for increasing intramuscular creatine stores leading to greater improvements in muscle mass, performance and recovery compared to placebo [2]. While effective, these non-loading creatine supplementation dosing strategies (Figure 1, side B) delay maximum intramuscular creatine storage. For example, in the classic ‘loading’ vs. daily ‘maintenance’ dose comparison study by Hultman et al. [35], creatine accumulation in muscle was similar (~ 20% increase) after participants consumed 3 g/day for 28 days or 20 g/day for 6 days [35]. Thus, it is currently recommended that individuals consume ~3-5 g/day of creatine for a minimum of 4 weeks in order to experience similar skeletal muscle saturation levels. Determination of which creatine supplementation strategy is preferred may depend on the goal of the individual. For instance, if an athlete is hoping to maximize the ergogenic potential of creatine supplementation in a very short period of time (< 30 days), adopting the creatine ‘loading’ strategy may be advised. However, if an athlete or exercising individual is planning to ingest creatine over an extended period of time (> 30 days), or if avoiding potential weight gain which can sometimes occur during creatine ‘loading’, the creatine ‘maintenance’ strategy would be a viable option. Athletes who are carrying out a creatine loading phase (i.e., 20 g/day) should emphasize the smaller dosing strategies (e.g. less than or equal to 10 gram servings) throughout the day, as dosages of greater than 10 grams may potentially lead to gastrointestinal distress (i.e., diarrhea) [105].

***In summary, accumulating evidence indicates that you do not have to ‘load’ creatine. Lower, daily dosages of creatine supplementation (i.e. 3-5 g/day) are effective for increasing intramuscular creatine stores, muscle accretion and muscle performance/recovery.***

### Is creatine beneficial for older adults?

There has been an increasing number of studies showing that creatine supplementation plays a therapeutic role in a variety of clinical conditions (see Gualano et al. [106] for a comprehensive review on this topic).

Perhaps one of the most promising conditions that could benefit from creatine supplementation is age-related sarcopenia. Sarcopenia is defined as a progressive and generalized skeletal muscle condition (i.e. decrease in muscle mass, strength, and functionality) that is associated with increased likelihood of adverse outcomes including falls, fractures, physical disability and mortality [107]. While resistance training is considered cornerstone in the treatment of sarcopenia [108], accumulating evidence indicates that creatine supplementation may enhance the anabolic environment produced by resistance training, subsequently mitigating indices of sarcopenia [9, 10, 19, 27].

Creatine supplementation can increase functionality (e.g., strength, activities of daily living, delay fatigue) and muscle mass in older adults [9, 10, 19, 87, 95, 109, 110]. However, the literature indicates that creatine alone (that is, without a concomitant resistance training program) is unlikely to result in substantial gains in muscle strength and functional performance [95, 111–113], although it does improve some parameters of muscle fatigue [114–116]. Likewise, most studies failed to show a beneficial effect of chronic creatine supplementation alone (≥ 30 days) on lean mass [98, 99, 113, 114]. For instance, we recently showed that creatine supplementation was not able to increase lean mass in postmenopausal women who supplemented with creatine (3 g/day) for 2 years, suggesting that creatine supplementation without exercise may be ineffective to prevent sarcopenia [99]. It is likely that increases in lean mass occasionally attributed to creatine supplementation in short-term studies (e.g., 7 days) are explained by increased body water, since creatine is osmotically active and it can sometimes induce water retention.

Conversely, substantial evidence indicates that creatine supplementation is capable of augmenting the hypertrophic response to resistance training in young adults [117], which is extended to older adults, as confirmed by three systematic reviews and meta-analyses [19, 118, 119]. The fact that creatine is more effective when combined with a training stimulus suggests that the main mechanistic action of creatine is its ability to enhance training volume and/or intensity, which may influence muscle protein kinetics, growth factors, satellite cells, inflammation and/or oxidative stress [9, 10, 19], ultimately resulting in greater skeletal muscle adaptations.

Regarding aging bone, emerging research over the past decade has shown some benefits from creatine supplementation. For example, healthy older males (> 50 yrs) who supplemented with creatine and performed whole-body resistance training for 10-12 weeks experienced an increase in upper limb bone mineral content [120] and a reduction in bone resorption compared to placebo [121]. More recently, Chilibeck et al. [122] showed that 52 weeks of creatine supplementation and supervised whole-body resistance training attenuated the rate of bone mineral loss in the hip region compared to placebo in postmenopausal females. However, a 2 year creatine supplementation protocol was infective for improving bone mass or bone geometry in post-menopausal women, again suggesting that creatine should be combined with resistance-type exercise to produce beneficial bone adaptations [99].

From a clinical and healthy aging perspective, it is recommended that creatine supplementation be combined with resistance training to produce the greatest adaptations in older adults. Future clinical trials involving frail populations with long-term follow-up(s) and larger samples are needed. The therapeutic potential of creatine supplementation for cachexia, myopathies, post-surgery rehabilitation, bed rest, other muscle/bone wasting condition/diseases and brain health warrants further investigation.

***In summary, there is growing body of evidence showing that creatine supplementation, particularly when combined with exercise, provides musculoskeletal and performance benefits in older adults.***

### Is creatine only useful for resistance / power type activities?

Although creatine supplementation has been theorized to primarily benefit athletes involved in high-intensity intermittent resistance/power type activities, there is a growing body of evidence suggesting that creatine supplementation may also provide beneficial effects for other activities. For example, creatine supplementation with carbohydrate [123] or carbohydrate and protein [124] has been reported to promote greater muscle glycogen storage than carbohydrate supplementation alone. Since glycogen replenishment is important for promoting recovery and preventing overtraining during intensified training periods [2, 125], creatine supplementation may help athletes who deplete large amounts of glycogen during training and/or performance (i.e., sporting events) to maintain optimal glycogen levels. Second, there is evidence that creatine supplementation may reduce muscle damage and/or enhance recovery from intense exercise. For example, Cooke and colleagues [126] reported that creatine supplementation during recovery from exercise-induced muscle damage promoted less muscle enzyme efflux and better maintenance of isokinetic muscle performance. Moreover, there is evidence that individuals supplementing their diet with creatine experienced less muscle damage, inflammation, and muscle soreness in response to running 30-km [127] as well as during 4-weeks of intensified training [70]. Consequently, creatine supplementation may help athletes recover from intense exercise and/or tolerate intensified periods of training to a greater degree. Third, there is evidence that athletes who supplement with creatine during training experience fewer musculoskeletal injuries, accelerated recovery time from injury [78, 128] and less muscle atrophy after immobilization [129, 130]. Whether this is due to a greater resistance to injury and/or ability to recover from injury remains unclear. Fourth, creatine supplementation (with or without glycerol) has been reported to help athletes hyper-hydrate and thereby enhance tolerance to exercise in the heat [28, 37, 131–145]. Therefore, creatine supplementation may reduce the risk of heat related-illness when athletes train and/or compete in hot and humid environments [72, 146]. Finally, there is evidence from animal models that creatine supplementation is neuroprotective [147–149] and can reduce the severity of spinal cord injury [150, 151], cerebral ischemia [152–155], and concussion/traumatic brain injury [2, 7, 12, 22, 32, 33, 156]. This evidence was so compelling that the International Society of Sports Nutrition recommended that athletes engaged in sports that have a potential for concussion and/or spinal cord injury take creatine for its neuroprotective effects [2]. Thus, there are a number of reasons beyond the ergogenic benefit that all types of athletes may benefit.

***In summary, there is a variety of athletic events, not just resistance/power activities, which may benefit from creatine supplementation.***

### Is creatine only effective for males?

Creatine kinetics may vary between healthy males and females [157]. Females may have higher intramuscular creatine concentrations [158] possibly due to lower skeletal muscle mass [159]. Potentially, the higher resting intramuscular creatine concentration in females (based on the upper limit of intramuscular creatine storage) may help explain some research showing diminished responsiveness and/or performance effects on females [160, 161].

As a result of hormone-driven changes in endogenous creatine synthesis, creatine transport, and creatine kinase (CK) kinetics, creatine bioavailability throughout various stages of female reproduction is altered, highlighting the potential positive implications for creatine supplementation in females [29]. The implications of hormone-related changes in creatine kinetics has been largely overlooked in performance-based studies [29]. Specifically, creatine supplementation may be of particular importance during menses, pregnancy, post-partum, perimenopause and postmenopause. Creatine kinase, as well as enzymes associated with creatine synthesis, are influenced by estrogen and progesterone [1]. Creatine kinase levels are significantly elevated during menstruation [162], with CK levels decreasing throughout the menstrual cycle, pregnancy, and with age. The lowest range of CK values have been reported during early pregnancy (20 weeks or less), equating to about half the concentration found at peak levels (teenage girls) [162, 163].

Maternal creatine supplementation during pregnancy in pre-clinical animal studies have demonstrated a protective effect against fetal death and organ damage associated with intrapartum hypoxia [164, 165]. Reduced creatine levels in late pregnancy have also been associated with low fetal growth [165]. There is additional data that metabolic demand from the placenta during gestation further lowers the creatine pool of the mother [166], which may be associated with low birth weight and pre-term birth. Creatine supplementation during pregnancy has been shown to enhance neuronal cell uptake of creatine and support mitochondrial integrity in animal offspring, thereby reducing brain injury induced by intrapartum asphyxia [167, 168]. Although there are no human studies evaluating the effects of creatine supplementation during pregnancy, creatine could provide a safe, low-cost nutritional interventional for reducing intra- and post-partum complications associated with cellular energy depletion [169]. This may be more important if the female is vegetarian, or unable to consume meat due to nausea or taste preferences (i.e. meat contains about 0.7 g of creatine/6 oz serving [54];).

Females have been reported to have lower levels of creatine in the brain (frontal lobe) [170]. Increasing creatine concentrations in the brain as a result of supplementation, particularly in females, may support the reported benefits of reducing symptoms of depression [171, 172] and ameliorating the effects of traumatic brain injury [12, 22]. Depression is about 2 times higher among females throughout the reproductive years [173] and accelerates around pubertal hormonal changes [174]. Altered brain bioenergetics and mitochondrial dysfunction have been linked with depression, particularly as it relates to CK, ATP, and inorganic phosphate (P_i_). Creatine supplementation has been shown to significantly augment cerebral PCr and P_i_ [175], particularly in females. The increase in cerebral PCr from 10 g of creatine supplementation was reported to be inversely related to symptoms of depression in adolescent females resistant to selective serotonin reuptake inhibitors [171] It appears that creatine supplementation may be effective for supporting creatine kinetics, mood, and pregnancy/fetal outcomes.

There is a small body of research that has investigated the effects of creatine supplementation in younger females. For example, Vandenberghe et al. [176] showed that creatine supplementation (20 g/day for 4 days followed by 5 g/day thereafter) during 10 weeks of resistance training significantly increased intramuscular concentrations, muscle mass and strength compared to placebo in females (19-22 yrs). In elite female soccer players (22 ± 5 yrs), creatine supplementation (20 g/day for 6 days) improved sprint and agility performance compared to placebo [177]. Hamilton et al. [178] showed that creatine supplementation (25 g for 7 days) augmented upper-body exercise capacity in strength-trained females (21-33 yrs) compared to placebo (19-29 yrs). Furthermore, in college-aged females (20 yrs), creatine supplementation (0.5 g/kg of fat-free mass for 5 days) improved knee extension muscle performance compared to placebo [179]. In contrast, not all data show improved performance in females [89, 160, 161]. Additionally, Smith-Ryan et al. [180] reported no significant effects of creatine loading on neuromuscular properties of fatigue in young adult females. It is important to evaluate the benefit to risk ratio; as noted elsewhere in this document, there are minimal risks associated with creatine supplementation, particularly when it is evaluated against the potential benefits in females.

Accumulating research over the past decade in postmenopausal females demonstrates that creatine supplementation during a resistance training program can improve muscle mass, upper- and lower-body strength, and tasks of functionality (30-s chair stand, lying prone-to-stand test, arm curl test) (for detailed review see Candow et al. [9]). Creatine supplementation appears to be a viable option for post-menopausal females to improve muscle quality and performance. In addition to its beneficial effects on aging muscle, creatine supplementation may also have favorable effects on bone in postmenopausal females, if combined with resistance training. For example, postmenopausal females who supplemented daily with 0.1 g/kg/day of creatine during 52-weeks of supervised whole-body resistance training experienced an attenuation in the rate of bone mineral loss at the femoral neck (hip), compared to females on placebo during training [122]. Furthermore, 5 g/day of creatine supplementation during 12 weeks of resistance training in postmenopausal females resulted in a significant increase in muscle mass and upper- and lower-body strength, compared to placebo [181]. However, even without the stimulus of resistance training, there is some evidence that creatine supplementation can still be beneficial. For example, in aging females (n=10; 67 ± 6 yrs), acute creatine supplementation (0.3 g/kg/day for 7 days) significantly improved lower-extremity physical performance (sit-to-stand test) [110], and fat-free mass and upper- and lower-body strength compared to placebo [86].

***In summary, there is accumulating evidence that creatine supplementation has the potential to be a multifactorial therapeutic intervention across the lifespan in females, with little to no side effects.***

### Are other forms of creatine similar or superior to monohydrate and is creatine stable in solutions/beverages?

Creatine monohydrate powder has been the most extensively studied and commonly used form of creatine in dietary supplements since the early 1990s [2, 125]. Creatine monohydrate was used in early studies to assess bioavailability, determine proper dosages, and assess the impact of oral ingestion of creatine on blood creatine and intramuscular creatine stores [35, 60, 182]. These studies indicated that orally ingested creatine monohydrate (e.g., 3–5 g/day) increases blood concentrations of creatine for 3-4 hours after ingestion thereby facilitating the uptake of creatine into tissue through diffusion and creatine transporters [1, 183, 184]. Additionally, it is well established that ~99% of orally ingested creatine monohydrate is either taken up by tissue or excreted in the urine as creatine through normal digestion [60, 185, 186]. Short-term loading with creatine monohydrate (e.g., consuming 5 g, 4 times daily for 5-7 days) has been reported to increase intramuscular creatine stores by 20–40% and exercise performance capacity by 5–10% [2, 125]. Creatine monohydrate supplementation during training (e.g., 5–25 g/day for 4–12 weeks) has been reported to promoted gains in muscle mass, strength, and exercise capacity [2, 125]. Despite the known efficacy, safety, and low cost of creatine monohydrate; a number of different forms of creatine have been marketed as more effective with fewer anecdotally reported adverse effects [187]. These marketing efforts have fueled speculation that creatine monohydrate is not the most effective or safest form of creatine to consume. This notion is clearly refuted by understanding the well-known physio-chemical properties of creatine monohydrate, as well as current creatine supplementation literature.

A number of different forms of creatine (e.g., creatine salts, creatine complexed with other nutrients, creatine dipeptides, etc.) have been marketed as more effective sources of creatine than creatine monohydrate [187]. However, there are no peer-reviewed published papers showing that the ingestion of equal amounts of creatine salts [188–191] or other forms of creatine like effervescent creatine [128], creatine ethyl ester [43, 192, 193], buffered creatine [41], creatine nitrate [194, 195], creatine dipeptides, or the micro amounts of creatine contained in creatine serum [196] and beverages (e.g., 25–50 mg) increases creatine storage in muscle to a greater degree than creatine monohydrate [187]. In fact, most studies show that ingestion of these other forms have less physiological impact than creatine monohydrate on intramuscular creatine stores and/or performance and that any performance differences were more related to other nutrients that creatine is bound to or co-ingested with in supplement formulations. This makes sense given that these other forms contain less creatine per gram than creatine monohydrate and that 99% of ingested creatine monohydrate is absorbed into the blood, then taken up into muscle, or excreted in urine [187].

Creatine monohydrate crystallizes from water as monoclinic prisms that hold one molecule of water of crystallization per molecule of creatine [187]. Subsequent drying of creatine monohydrate at about 100°C removes the water of crystallization yielding anhydrous creatine (100% creatine) [187]. Creatine is considered a weak base (pKb 11.02 at 25°C) that can only form salts with strong acids (i.e., pKa < 3.98). Creatine can also serve as a complexing agent with other compounds via ionic binding. Creatine monohydrate powder contains the highest percentage of creatine (87.9%) other than creatine anhydrous [187]. Creatine monohydrate manufactured in Germany involves adding acetic acid to sodium sarconsinate, heating, adding cyanamide, cooling to promote crystallization, separation and filtration, and drying has been reported to produce 99.9% pure creatine monohydrate with no contaminants. Meanwhile, other sources of creatine monohydrate that have different starting materials (e.g., sarcosinates and O-alkylisourea, sarcosinates and S-alkylisothiourea) and methods of creatine synthesis, particularly from sources produced in China, have been found to contain up to 5.4% dicyandiamide, 0.09% dihydrotriazine, 1.3% creatinine, dimethyl sulphate, thiourea, and/or higher concentrations of heavy metals like mercury and lead due to use of different chemical precursors, poorly controlled synthesis processes, and/or inadequate filtration methods that more readily produce these contaminants [197]. While the effects of ingesting these compounds on health are unknown, contamination with dihydrotriazine has been suggested to be of greatest concern since it is structurally related to carcinogenic compounds [197]. For this reason, German sourced creatine monohydrate has been primarily used in research to establish safety and efficacy and is therefore the recommended source of creatine monohydrate to use in dietary supplements [2, 187].

Creatine monohydrate powder is very stable showing no signs of degradation into creatinine over years, even at elevated storage temperatures [187]. However, creatine is not stable in solution due to intramolecular cyclization that converts creatine to creatinine especially at higher temperatures and lower pH [187, 198–200]. The degradation of creatine can be reduced or halted by lowering the pH under 2.5 or increasing the pH above 12.1 [187]. This is the reason that less than 1% of creatine monohydrate is degraded to creatinine during the digestive process and creatine is taken up by tissue or excreted in urine after ingestion [60, 185–187]. Moreover, since creatine is an ampholytic amino acid, it is not very soluble in water (e.g., creatine monohydrate dissolves at 14 g/L at 20°C with a neutral pH of 7) [187]. Mixing creatine in higher temperature solution increase solubility, which is the reason why initial studies administered creatine in hot tea [35, 60, 103, 104, 123, 182] but the solubility has no influence on tissue uptake [187]. The lack of solubility and stability of creatine in solution is the reason that creatine is primarily marketed in powder form and efforts to develop stable beverages containing physiologically effective doses of creatine (e.g., 3–5 g per serving) have been unsuccessful.

***In summary, while some forms of creatine may be more soluble than creatine monohydrate when mixed in fluid, evidence-based research clearly shows creatine monohydrate to be the optimal choice.***

## Conclusions

Based on our evidence-based scientific evaluation of the literature, we conclude that:

1. Creatine supplementation does not always lead to water retention.
2. Creatine is not an anabolic steroid.
3. Creatine supplementation, when ingested at recommended dosages, does not result in kidney damage and/or renal dysfunction in healthy individuals.
4. The majority of available evidence does not support a link between creatine supplementation and hair loss / baldness.
5. Creatine supplementation does not cause dehydration or muscle cramping.
6. Creatine supplementation appears to be generally safe and potentially beneficial for children and adolescents.
7. Creatine supplementation does not increase fat mass.
8. Smaller, daily dosages of creatine supplementation (3-5 g or 0.1 g/kg of body mass) are effective. Therefore, a creatine ‘loading’ phase is not required.
9. Creatine supplementation and resistance training produces the vast majority of musculoskeletal and performance benefits in older adults. Creatine supplementation alone can provide some muscle and performance benefits for older adults.
10. Creatine supplementation can be beneficial for a variety of athletic and sporting activities.
11. Creatine supplementation provides a variety of benefits for females across their lifespan.
12. Other forms of creatine are not superior to creatine monohydrate.

## Data Availability

Not applicable.

## Abbreviations

ACSM: American College of Sports Medicine
ATP: Adenosine triphosphate
C: Celsius
CK: Creatine kinase
CSA: Controlled substances act
DEA: Drug enforcement association
DHT: Dihydrotestosterone
DSHEA: Dietary Supplement Health and Education Act
ECW: Extracellular water
FDA: Food and Drug Administration
G: Grams
GMP: Good Manufacturing Practices
ICW: Intracellular water
ISSN: International Society of Sports Nutrition
Kg: Kilogram
Km: Kilometer
L: Liter
MPS: Muscle protein synthesis
NCAA: National Collegiate Athletic Association
Nmol: Nanomole
Oz: Ounce
PCr: Phosphocreatine
pH: Potential hydrogen
s: Seconds
pKa: Acid dissociation constant
P_i_: Inorganic phosphate
TBW: Total body water
Yrs: Years of age
